# A rare occurrence of non-classic congenital adrenal hyperplasia and type 1 diabetes mellitus in a girl with Prader-Willi Syndrome: Case report and review of the literature

**DOI:** 10.3389/fendo.2023.1148318

**Published:** 2023-04-12

**Authors:** Alessia Aureli, Sarah Bocchini, Michela Mariani, Antonino Crinò, Marco Cappa, Danilo Fintini

**Affiliations:** ^1^ Prader-Willi Reference Center, Endocrinology Unit, Bambino Gesù Children Hospital, IRCCS, Rome, Italy; ^2^ Center for Rare Diseases and Congenital Defects, Fondazione Policlinico Universitario A. Gemelli, IRCCS, Rome, Italy

**Keywords:** Prader-Willi Syndrome, adrenarche, premature pubarche, pubertal development, growth hormone deficiency, congenital adrenal hyperplasia, diabetes mellitus

## Abstract

Prader–Willi syndrome (PWS) is a rare genetic disorder resulting from lack of expression of the paternally derived chromosome 15q11–13, associated with several complications, including pubertal disorders, short stature, hyperphagia, obesity, glucose metabolism abnormalities, scoliosis, obstructive sleep apnea syndrome (OSAS) and behavioral problems. We report the case of a girl affected by PWS who presented at the age of 5.9 with premature pubarche, accelerated linear growth and advanced bone age (BA). She was subsequently diagnosed with non-classic congenital adrenal hyperplasia (CAH) confirmed by genetic analysis. Considering the clinical, biochemical, and genetic findings, hydrocortisone therapy was started to prevent rapid BA acceleration and severe compromission of final height. During infancy, short stature and low levels of insulin-like growth factor-1 (IGF-1) for age and gender led to suspicion of growth hormone deficiency (GHD), confirmed by stimulation testing (arginine and clonidine). rhGH therapy was administered and continued until final height was reached. During endocrinological follow up she developed impaired glucose tolerance with positive markers of β-cell autoimmunity (anti-glutamic acid decarboxylase antibodies, GAD Ab), which evolved over time into type 1 diabetes mellitus and insulin therapy with a basal-bolus scheme and an appropriate diet were needed.

## 1 Introduction

### 1.1 Prader-Willi Syndrome

Prader-Willi Syndrome (PWS) is a rare disease and the most frequent form of syndromic obesity, with a prevalence rate of 1 in 10,000 to 30,000 live births (1, 2). This genetic disorder is caused by the absence of the paternally expressed imprinted genes in the critical region of chromosome 15 at the locus 15q11.2-q13, which can derive from paternal deletion (del15) (65-75% of individuals), maternal uniparental disomy (UPD15) (20-30%), or an imprinting defect (ID15) (1-3%) (1, 2).

PWS is characterized by a multitude of signs and symptoms, many of which possibly linked to hypothalamic dysfunction (3), including: neonatal hypotonia accompanied by failure to thrive, followed by hyperphagia in early childhood and gradual development of morbid obesity, dysmorphic features (characteristic facial appearance with turricephaly, small hands and feet, scoliosis and kyphosis), intellectual disability, behavioral problems and multiple endocrine abnormalities, including GH deficiency (GHD) and hypogonadism (2).

#### 1.1.1 Adrenarche and pubertal development in PWS

Early adrenarche and higher prevalence of premature pubarche (PP) have been described in PWS, although data on prevalence and age of onset are mostly based on case series (4). The estimated prevalence of PP in PWS is approximately 30% in girls and 16% in boys (4). Adrenarche is defined as the increase in production of adrenal androgens, most specifically dehydroepiandrosterone sulphate (DHEA-S) (5). It is a gradual process characterized by the appearance of adult body odor, acne and blackheads, oily hair and pubic and/or axillary hair (5) which usually occurs after the age of 8 years in girls and 9 years in boys. Early adrenarche and PP in children with PWS possibly result from earlier maturation of the zona reticularis of the adrenal glands, independently of hypothalamic-pituitary-adrenal axis activation (5, 6) which is heterogeneous in PWS. Nevertheless, PWS is characterised by a slower pubertal pace, with a higher age at Tanner stage P5 in both sexes. In girls a higher age at menarche with respect to the general population is described, whereas a minority may present with pubertal arrest and/or primary amenorrhea (7, 8). Males with PWS often present hypothalamic hypogonadism, although recently primary testicular dysfunction has been advocated as a major contributor to abnormal pubertal development (9, 10). Although puberty and sexual maturation are usually delayed or incomplete in PWS, rare occurrences of true precocious puberty are described (11, 12).

#### 1.1.2 Diabetes mellitus in PWS

Diabetes mellitus can affects PWS patients and it typically presents with a type 2 (T2DM) phenotype (13), with a reported prevalence of 7-24% (14–16). Nonetheless, rare cases presenting as diabetes type 1 (T1DM), or a monogenic diabetes (maturity onset diabetes of the young, MODY), have also been described (17–19). Furthermore, occasional reports of islet autoantibodies in diabetic PWS subjects were associated with a T2DM phenotype (20). In a group of 23 diabetic PWS patients with age at onset <20 years (13.4 ± 3.9 years), β-cell antibodies were found in 40% of subjects (21). Besides these scarce reports, islet autoantibodies and other markers of autoimmune islet cell destruction in PWS have not been extensively studied.

In PWS it has been traditionally assumed that abnormal glucose metabolism develops as a consequence of obesity. In accordance, non-obese subjects show lower insulin and glucose values compared to obese PWS individuals, both in children and in adults (22–24). Nevertheless, the relationship between obesity and the development of diabetes is not completely elucidated and may be different than in individuals with simple obesity.

### 1.2 Congenital adrenal hyperplasia

Congenital adrenal hyperplasia (CAH) represents a heterogeneous group of autosomal recessive disorders caused by deficient adrenal corticosteroid biosynthesis (25, 26). It arises from enzymatic defects in the adrenal steroidogenic pathway or in the electron-providing factor, (P450 oxidoreductase - POR). Between 90% and 95% of cases of CAH are caused by 21-hydroxylase deficiency (21OHD) encoded by the *CYP21A2* gene (25, 26) located on the short arm of chromosome 6 (6p21.3).

In Western societies, the incidence of classic 21OHD (present at birth with or without salt-wasting and cortisol deficiency) varies from 1 in 10.000-15.000 live births. Non-classic (or late-onset) 21OHD is more common, with an incidence of about 1 in 500 to 1.000 live births (27). The condition arises because of defective conversion of 17-hydroxyprogesterone (17OHP) to 11-deoxycortisol. Defective cortisol biosynthesis results in reduced hypothalamic-pituitary negative feedback and increased ACTH secretion; consequently, adrenal androgens are produced in excess. The genotype-phenotype correlation in CAH due to the 21OHD is well established and the clinical phenotype is dependent on residual enzymatic activity provided by the less severely mutated allele.

Several clinical variants have been identified from genital ambiguity in newborns girls to precocious pseudopuberty in both males and females. During adolescence and adulthood, females can present with a phenotype similar to polycystic ovary syndrome (PCOS) with hirsutism, primary or secondary amenorrhea, or anovulatory infertility (28). In the salt-wasting form the patients also present concomitant cortisol and aldosterone deficiency; it usually occurs after the first 2 weeks of life and neonates show hypotension, poor feeding, vomiting, lethargy, and sepsis-like symptoms.

The remaining 5-10% of CAH forms are caused by mutations in other steroidogenic enzymes involved in adrenal steroidbiosynthesis, such as: C*YP11B1*, C*YP17A1* and *HSD3B2*, encoding respectively 11β-hydroxylase, 17α-hydroxylase and 3β-hydroxysteroid dehydrogenase type 2.

Association between PWS and adrenal congenital hyperplasia is very rare and only sporadic case reports are described in literature (29, 30).

## 2 Case report

**Figure 1:**
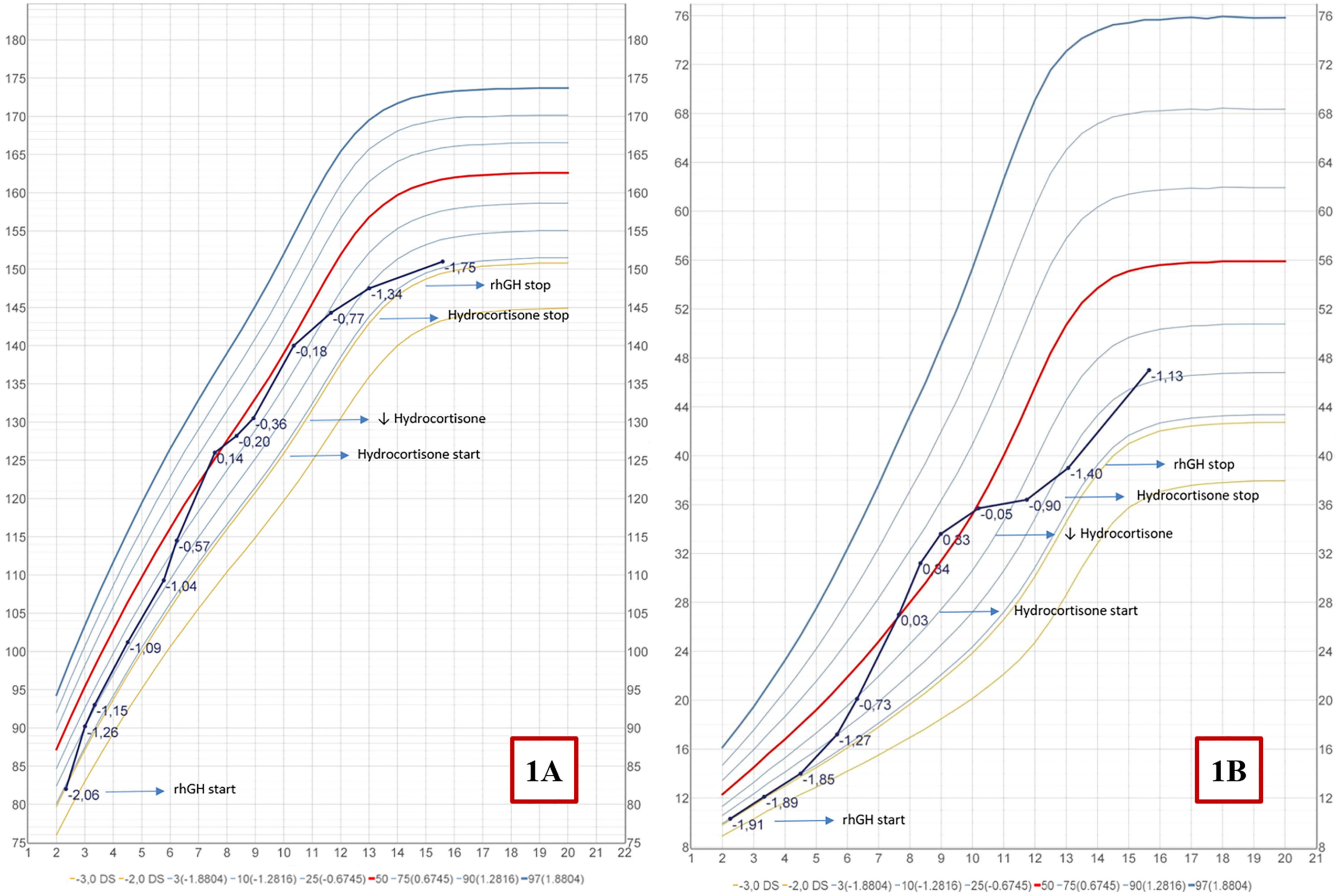
Italian growth chart for height **(A)** and weight **(B)** (34).

We report the case of C.B., a 16 years-old girl affected by PWS, who presented PP in the context of CAH by 21OHD and later developed T1DM.She was born at 37 weeks by cesarean section, her birth weight was 2500 g (-0,89 standard deviation scores [SDS]). During infancy she presented with severe hypotonia, respiratory distress, weak cry, small hands and feet, which led to the suspicion of PWS. At 12 months of age the diagnosis was confirmed by methylation-specific multiplex ligation-dependent probe amplification analysis (MS-MLPA) and chromosome 15 DNA polymorphism analysis conducted on the proband and her parents, demonstrated the presence of maternal UPD. During childhood she presented neurodevelopmental disorders comprising learning and intellectual disability, language delay and impairment. Neuropsychiatric evaluation did not report behavioral disorders, although skin picking of the four limbs was found on pre-existing injuries. Food seeking behavior was controlled through parental training, providing a strict schedule of meals and avoiding the intake of sweet and high calorie foods from infancy; this is particularly relevant since the hyperphagia phase in PWS usually occurs between 2 and 4 years of age, and is then followed by an abnormal interest towards food (31). At the age of 2.4 years she presented short stature (**Table 1**) alongside reduced levels of insulin-like growth factor-1 (IGF-1) for age and gender (31 ng/mL, n.v. 49-289) and growth hormone (GH) stimulation tests with arginine (0.5 g/Kg iv) and clonidine (150 µg/m^2^ orally) were performed, leading to the diagnosis of GHD (**Table 2**). According to the 2006 Italian Drug Agency (AIFA) Note 39 criteria (32) rhGH therapy was started at a dose of 0.033 mg/Kg/d (0.7 mg/m²/d); thereafter the dose was titrated according to IGF-1 levels, keeping it within <2 SDS, according to guidelines for GHD treatment in PWS (31, 33). Clinical follow up was established every six months and polysomnography were performed once a year to exclude obstructive sleep apnea syndrome (OSAS) which can worsen during rhGH therapy. A controlled diet with the support of a nutrition specialist permitted to control the patient’s body mass index (BMI) keeping it in the normal weight range (**Figure 1**). Regular growth with a height velocity (HV) of 6-7 cm/yr was registered until the age of 5.5 (75° centile; +0.74 SDS), alongside pre-pubertal Tanner stage 1 by physical examination. At 5.5 years of age pubic hair growth was noted, without thelarche, and at the age of 5.9 an endocrinological evaluation was requested (**Tables 1**, **2**). Six months later, elevated 17OHP and DHEA-S levels were confirmed, HV attested at 10.4 cm/yr (>97° centile; +4.7 SDS) and physical examination confirmed a Tanner stage Ph2-Pb1, with no evidence of axillary hair. Stimulation test for CAH with tetracosactide 250 µg IV was conducted showing basal and stimulated level of 17OHP >10 ng/mL, both diagnostic for 21OHD (35). The patient and her parents were referred for genetic testing (MLPA confirmed by Sanger sequencing) for non-classic CAH due to 21OHD with evidence of compound heterozygosity for *CYP21A2* mutation: V281L, inherited from the mother, and A(C)656G [intron 2], inherited from the father. At the age of 7.7 years the patient confirmed accelerated HV (10 cm/aa; + 5.2 SDS), physical examination revealed Tanner stage Ph3-Pb2, initial axillary hair and clitoral enlargement; bone age (BA) was advanced corresponding to 9.5 years, according to Greulich&Pyle method, and pelvic ultrasound showed infantile uterine morphometry, with maximum ovarian volume of 1.2 mL and a thin endometrium. The evidence of pubertal progression prompted the execution of a stimulation test with 100 µg IV gonadotropin-releasing hormone (GnRH test), which showed a FSH and LH peak in accordance with pubertal activation of the hypothalamic-pituitary-gonadal axis (**Table 2**). According with the biochemical and clinical evidence of hyperandrogenism which led to the linear growth acceleration, and considering the risk of premature epiphyseal closure, therapy with hydrocortisone 20 mg/m^2^/d was started in order to promptly suppress androgen levels. Eight months after the start of therapy, Tanner stage was Ph3-Pb2, suppressed levels steroid hormones were observed, HV declined to 5 cm/yr and a left-hand X-ray documented a BA of 10.5 years (**Tables 1**, **2**). An oral glucose tolerance test (oGTT 1.75 g/Kg) showed an impaired glucose tolerance (IGT), and therefore rhGH therapy and hydrocortisone therapy were reduced to 0.021 mg/Kg/d and 14 mg/m^2^/d, respectively). At the age of 9.0 the patient presented HV 3.9 cm/yr (3rd centile; -1.9 SDS) and Tanner stage did not change. Considering the patient having entered an appropriate age for pubertal start (>8 years-old), the HV and pubertal stage stability, hydrocortisone therapy was reduced to 11.5 mg/m^2^/d and rhGH therapy increased to 0.026 mg/Kg/d, to allow linear growth and spontaneous pubertal progression.

**Table 1:** Clinical features during endocrinological follow up.

Years	2,4^Δ^	5.9	6.3¤	7.7*	8.4	9^#^	15.7^
	-	-	-	-	-	-	-
Auxological parameters							
Height (cm)	81 (-2.16 SDS)	109.3 (-1.04 SDS)	114.5 (-0.57 SDS)	126 (+0.14 SDS)	128.2 (-0.2 SDS)	130.5 (-0.36 SDS)	151 (-1.75 SDS)
Weight (Kg)	10.5 (-1.73 SDS)	17.2 (-1.35 SDS)	20.1 (-0.68 SDS)	27 (+0.06 SDS)	31.2 (+0.33 SDS)	33.6 (+0.35 SDS)	47 (-1.13 SDS)
BMI (Kg/m^2^)	16 (+0.04 SDS)	14.1 (-1 SDS)	15.3 (-0.45 SDS)	17 (+0.16 SDS)	19 (+0.7 SDS)	19.8 (+0.8 SDS)	20.6 (-0.15 SDS)
HV (cm/yrs)	5.6 (-2.03 SDS)	6.5 (+0.33 SDS)	10.4 (+4.7 SDS)	10 (+5.2 SDS)	5 (-0.63 SDS)	3.9 (-1.9 SDS)	< 2
Pubertal Stage							
Breast	1	1	1	2	2	2	3
Pubic hair	1	2	2	3	3	3	5

^Δ^rhGH was started (0,33mg/Kg·d); **¤**Patient and her parents were referred for genetic testing for non-classic CAH; *Hydrocortisone was started (20 mg/m^2^); #Hydrocortisone was reduced (11.5 mg/m^2^) and rhGH was increased; ^Hydrocortisone and rhGH were gradually reduced and stopped at the ages of 11.8 and 14.7 years, respectively.

BMI, body mass index; CAH, congenital adrenal hyperplasia; HV, height velocity; SDS, standard deviation score.

**Table 2:** Hormonal and radiological exams during endocrinological follow up.

Years	2.4^Δ^	5.9	6.3¤	7.7*	8.4	9^#^	15.7^
	-	-	-	-	-	-	-
Hormonal exams							
LH (mUI/mL)	-	<0.2	-	0.1	-	-	0.7
FSH (mUI/mL)	-	<0.2	-	3.4	-	-	6.1
17OHP (ng/mL)	-	7	10.7	14.9	0.1	-	-
DHEA-S (ng/mL)	-	971	2420	2040	<150	-	-
Δ4 (ng/mL)	-	-	1.44	-	<0.3	-	-
Te (ng/dL)	-	30	58.9	104	37.7	-	-
E2 (pg/mL)	-	42.9	-	28.3	17	-	23.5
ACTH (pg/mL)	-	24.7	-	25.2	-	-	-
Cortisol (mcg/ dL)	-	11.2	-	10.1	-	-	-
TSH (mU/L)	6.14	2.85	5.26	2.8	-	-	4.13
FT4 (ng/dL)	0.93	1.24	1.08	1.02	-	-	1.28
IGF-1 (ng/mL)	31	296	283	381	561	350	192
ACTH Tests							
17OHP (ng/mL) – 0’	-	-	10.7	-	-	-	-
17OHP (ng/mL) – 60’	-	-	14.9	-	-	-	-
GH Test							
Arg. GH (ng/mL) - Peack	2.9	-	-	-	-	-	-
Clon. GH (ng/mL) - Peack	3.88	-	-	-	-	-	-
GHRH + Arg. GH (ng/mL)- Peack	-	-	-	-	-	-	14.4
LHRH Test							
LH (mUI/mL) – Basal	-	-	-	0.1	-	-	-
LH (mUI/mL) – Peack	-	-	-	7.53	-	-	-
FSH (mUI/mL) – Basal	-	-	-	3.4	-	-	-
FSH (mUI/mL) – Peack	-	-	-	13.24	-	-	-
Radiological exams							
BA	1.3	-	7.1	9.5	10.5	11	-

^Δ^rhGH was started (0.33mg/Kg·d); **¤**Patient and her parents were referred for genetic testing for non-classic CAH; *Hydrocortisone (20 mg/m^2^) was started; #Hydrocortisone was reduced (11.5 mg/m^2^) and rhGH was increased; ^Hydrocortisone and rhGH were gradually reduced and stopped at the age of 11.8 and 14.7 years, respectively.

17OHP, 17-hydrossiprogesterone; CAH, congenital adrenal hyperplasia; DHEA-S, dehydroepiandrosterone; Δ4, delta-4-androstenedione; Te, testosterone; E2, estradiol; BA, bone age.

At this time the patient first presented a positive autoimmune screening for glucose metabolism disorders, with an anti-glutamic acid decarboxylase (GAD) titer of 54 U/mL (n.v. <0.9), with normal levels of fasting glucose and insulin.

During the follow-up hydrocortisone therapy was gradually reduced and then stopped at the age of 11.8. rhGH therapy was continued following IGF-1 levels and HV, gradually increased up to 0.034 mg/Kg/d and then stopped when final stature was reached at the age of 14.7 years (HV <2cm/yr). After five months from the stop of rhGH therapy, a retesting with Growth Hormone Releasing Hormone (GHRH) + arginine was performed and persistent GHD was excluded according to the current Italian Drug Agency (AIFA) Note 39 criteria (36). During the endocrinological evaluation at the age of 15.7 the patient presented: height 151 cm (-1.76 SDS), weight 47 kg (-1.13 SDS), BMI 20.6 (-0.15 SDS), Tanner stage Ph5-Pb3; biochemical and hormonal exams showed a mature hypothalamus-pituitary-gonadal axis (HPG) (**Table 2**), C-peptide attested at 1.14 ng/mL (in the lower part of reference range) and the IGT condition was confirmed.

At the age of 16.2 she presented with polyuria and polydipsia, weight loss and a fasting Hemoglucotest (HGT) >200 mg/dL. She referred to the emergency room, where she presented glucose levels of 382 mg/dL, HbA1c of 13.7%, C-peptide of 0.41 ng/mL, Na 141 mEq/L, K 4.8 mEq/L, glycosuria and ketonuria and an arterial blood gas analysis documented metabolic acidosis: pH 7.29, base excess -5.9, HCO3- 20.7 mmol/L, lactate 1.3 mmol/L, anion gap 19.3 mEq/L. Anti-insulin (IAA), anti-tyrosine phosphatase (IA2) and anti-ZnT8 antibodies were undetectable, whereas anti-GAD rose to 191 U/mL. Other exams showed normal thyroid function although with positive anti-thyroglobulin (Ab-Tg) and thyroid peroxidase antibodies (Ab-TPO), and a negative screening for celiac disease. Diagnosis of T1DM was made, insulin therapy with a basal-bolus scheme and an appropriate diet was started. The patient is currently followed at our Endocrine Center with a specific therapeutic program consisting in clinical and biochemical follow-up.

Written informed consent was obtained from the parents for the publication of this case report including clinical, biochemical and radiological data.

## 3 Discussion

Early adrenarche and higher prevalence of PP have been described in patients with PWS and in some of these cases a CAH diagnosis might be present (37). One study conducted on 120 PWS children between the ages of 2 and 17 years, described earlier adrenarche, with serum DHEA-S levels significantly higher in PWS girls and boys compared to healthy controls in the age groups from 3 to 10 years (37). Median age at onset of pubarche (Tanner stage Ph2) in children with PWS was 9.0 years in girls and 10.3 years in boys (37).

Our patient presented adrenarche and pubarche at the age of 5.5 years. In contrast with the literature, she showed fast progression in pubic hair growth; biochemical evaluation demonstrated a very high level of adrenal androgens, including 17OHP. At the first endocrinological evaluation the oestradiol (E2) concentration was also elevated, although with prepubertal levels of LH and FSH, which was interpreted as a condition of pseudopuberty due to excess of androgens and their consequent aromatization. For this reason, standard dose ACTH-stimulation test for 17OHP was performed.

PWS is a rare disease (estimated prevalence of 1:10.000-30.000) (1, 2), whereas CAH in its non-classic form is quite more common (1 in 500 to 1.000 live births in western countries) (27); as such their association may sporadically occur in the population. Nonetheless, the presence of both conditions is rare and to our knowledge only two cases are described in literature (29, 30). Patients with the non-classic form of CAH may present in early childhood with sexual precocity, pubic hair development or growth acceleration due to premature androgen excess. If left untreated, the sex steroid production stimulates premature epiphyseal closure, and final adult height is invariably reduced (38, 39). Additionally, central precocious puberty may develop in patients with CAH, possibly due to androgen activation of the HPG axis, thus exacerbating premature epiphyseal fusion (40, 41). Glucocorticoid therapy remains the standard treatment for patients with CAH. Some studies report a significant improvement in final adult height outcome in CAH children who are treated with GH, alone or in combination with GnRH analogues (42–44). rhGH therapy may counter the deceleration in growth velocity often associated with glucocorticoid therapy, whereas GnRH analogues suppress the HPG axis to prevent premature epiphyseal closure. However, since the addition of GnRH analogues has not been clearly shown to benefit GH therapy in increasing height gain, their use should be based on the age of the patient and the social impact of precocious puberty. In our patient genetic testing for 21OHD was performed with confirmation of non-classic CAH and as such hydrocortisone therapy was started. The use of corticosteroid therapy was decided based on advanced bone age, biochemical hyperandrogenism and increasing growth velocity to prevent rapid BA acceleration and severe compromission of final height, a common feature of PWS worsened by CAH (29, 44). The very high levels of adrenal androgens in our patient led us to the decision to start oral hydrocortisone at a dosage (20 mg/m^2^/d) above the recommended dose interval for non-classic CAH (10-15 mg/m^2^/d) (45) in order to suppress adrenal hyperandrogenism and prevent the effects of androgen excess on final height.

Our patient underwent clonidine and arginine GH stimulation testing, which revealed a condition of GHD (46). rhGH therapy was started at the age of 2.7 years within the recommended dose range for PWS. The prevalence of GHD in PWS is reported around 50%, nevertheless rhGH therapy produces several benefits in these subjects: increase in final height, improved body composition, muscle thickness, bone mineral density, cognitive function and behavior in PWS subjects (31, 47–49). Furthermore, rhGH therapy also influences sexual development due to the existence of a crosstalk between the HPG and the somatotropic axes (50) during development from infancy through puberty, transition age and, finally, in adult life (51–55).

Our patient did not show advanced clinical pubertal features and the pelvic ultrasound examination documented an infantile uterine feature, which prompted to avoid GnRH analogue therapy.

Autoimmune diseases are not described as a typical feature of PWS. Although thyroid dysfunction may also be observed in the syndrome, this is not the result of thyroid autoimmunity (56, 57). Nevertheless, an increased systemic low-grade inflammation was described in these patients. One paper observed that PWS subjects present overactivation of the innate immune system, independent of central adiposity and insulin-resistance (58). Among the factors that may influence this phenomenon, there are the GHD and hypogonadism described in PWS. Another frequent comorbidity in PWS patients is OSAS which may also contribute towards increased low-grade inflammation (59, 60). Nevertheless, despite their severe obesity, lower visceral fat mass, hypoinsulinemia and increased insulin sensitivity have been described in PWS patients by most (61, 62), although not all studies (22–24, 63), compared to non-syndromic obese patients (61, 62). According to early reports, lower fasting insulin levels and reduced insulin resistance (measured by HOMA-Index) have been reported at all ages in obese patients with PWS (64–66). A familial component in insulin resistance, as in the general population, could also contribute to the individual subject affected by PWS whereas the different genotypes (*del* or UPD15) do not appear to influence the development of altered glucose homeostasis in PWS (16, 63). Data about insulin secretion in PWS, however, are still under debate.

In our patient we observed persistent IGT although with a normal body weight, which led to a reduction of rhGH and hydrocortisone dosages. An initial improvement in glucose homeostasis was followed at the age of 9 by a positive autoimmune screening for anti-GAD with normal fasting glucose and insulin levels. At the age of 16.2 years the typical symptoms of diabetes mellitus emerged and diabetic ketoacidosis occurred as typical of type 1 phenotype (T1DM); concurrently she presented anti-thyroid antibodies, but a negative antibody screening for celiac disease. Despite the relationship between obesity and the development of diabetes being unclear in PWS, in our case it was most probable that the patient presented a genetic predisposition for autoimmune diseases, although in the absence of family history.

In conclusion, the present is the third case report of a subject with CAH associated with PWS, but the first confirmed by genetic analysis. Although CAH is a common disease, and PP a common feature in PWS, 17OHP measurement should be performed in all PWS children experiencing PP and early adrenarche to exclude a possible concurrent 21OHD. Furthermore, although PWS is most typically associated with a T2DM phenotype, anti-islet antibodies and C-peptide levels should be assayed at the occurrence of glucose metabolism impairments in children and adolescents affected by PWS to timely diagnose the sporadic T1DM cases and guide follow-up concerning disease progression and the need for insulin therapy.

## Data availability statement

The original contributions presented in the study are included in the article/supplementary material. Further inquiries can be directed to the corresponding author.

## Ethics statement

Ethical review and approval was not required for the study on human participants in accordance with the local legislation and institutional requirements. Written informed consent was obtained from the parents for the publication of this case report including clinical, biochemical and radiological data.

## Author contributions

AA collected data, drafted the initial manuscript, and critically reviewed and revised the manuscript. SB and MM contributed to the original draft of the manuscript. AC and MC critically reviewed and revised the manuscript for important intellectual content. DF coordinated and supervised data collection and manuscript drafting and revised the manuscript for intellectual content. All authors approved the final manuscript as submitted and agree to be accountable for all aspects of the work. 
